# Ultrasound diagnosis of a pseudoaneurysm of the internal right mammary artery

**DOI:** 10.1007/s40477-024-00889-6

**Published:** 2024-04-17

**Authors:** Andrea Boccatonda, Marco Balletta, Damiano D’Ardes, Giulio Cocco, Fabio Piscaglia, Carla Serra, Susanna Vicari, Cosima Schiavone

**Affiliations:** 1 Internal Medicine, Bentivoglio Hospital, AUSL Bologna, Via Marconi 35, Bentivoglio, Bologna Italy; 2 Department of Medical and Surgical Sciences, University of Bologna, Bologna, Italy; 3 Internal Medicine, IRCCS Azienda Ospedaliero-Universitaria Policlinico di Sant’Orsola, Bologna, Italy; 4 Department of Medicine and Aging Science, Institute of “Clinica Medica”, “G. d’Annunzio” University of Chieti, 66100 Chieti, Italy; 5 Internistic Ultrasound Unit, SS Annunziata Hospital, “G. D’Annunzio” University, 66100 Chieti, Italy; 6 Division of Internal Medicine, Hepatobiliary and Immunoallergic Diseases, IRCCS Azienda Ospedaliero-Universitaria di Bologna, Bologna, Italia; 7 Interventional, Diagnostic and Therapeutic Ultrasound Unit, IRCCS, Azienda Ospedaliero-Universitaria di Bologna, Bologna, Italy

**Keywords:** Trauma, Bleeding, Hematoma, Pseudoaneurysm, Mammary artery, Lung

## Abstract

One of the principles of managing trauma patients is that of their continuous re-evaluation over the hours and days. Even if the execution of the computed tomography method is classically recommended, especially in the most serious cases and in polytraumas with major dynamics, the clinician can use or request an ultrasound examination, especially in subsequent re-evaluations. Here we report a clinical case demonstrating how an ultrasound re-evaluation after the acute event can lead to a correct diagnosis of a rare complication of thoracic trauma. The findings were suggestive for a pseudoaneurysm of the internal right mammary artery. Subsequently, an ultrasound-guided injection of thrombin was carried out until complete interruption of the flow within the formation. At subsequent follow-up, no arterial or venous blush was highlighted.

**Supplementary Information:**

The online version contains supplementary material available at 10.1007/s40477-024-00889-6.

## Introduction

Ultrasound is a valuable method for evaluating a patient in an emergency setting [1–3]. Specifically, the FAST method has been developed in the field of trauma emergencies, based on 4 abdominal scans, with the aim of identifying signs of hemoperitoneum [4]. Over the years, the method has developed into E-FAST, providing six total scans also integrating lung assessments both at the bases (searching hemothorax) and in the right and left parasternal areas (searching pneumothorax) [5, 6].

This method therefore allows to identify and then treat rapidly life-threatening diseases, such as pneumothorax, cardiac tamponade and hemoperitoneum [7]. Moreover, there is the so-called “secondary survey” in the trauma patient, which consists of a “head to neck” evaluation aiming to identify any alteration at the level of the various skeletal segments and organs [8]. Even if the execution of the CT method is classically recommended, especially in the most serious cases and in polytraumas with major dynamics, the clinician can use or request an ultrasound examination, especially in subsequent re-evaluations [8].

Indeed, one of the principles of managing the trauma patients is that of their continuous re-evaluation over the hours and days. An example is the study of rib fractures, which presents excellent visibility on ultrasound while in some cases the finding is not clearly visible on x-ray and CT scan [9].

Here we report a clinical case demonstrating how an ultrasound re-evaluation after the acute event can lead to a correct diagnosis of a rare complication of thoracic trauma.

## Case presentation

A 49-years old male patient was hospitalized for trauma to the right hemithorax following an accidental fall at home. His past medical history was characterized by drug addiction, C hepatitis virus-related chronic liver disease, previous hospitalization for spondilodiscitis. He made a first access to the emergency room, where a chest X-ray was performed showing suspected right pulmonary contusion and IX right rib fracture. Subsequently, he was discharged with analgesic therapy prescription.

**Fig. 1:**
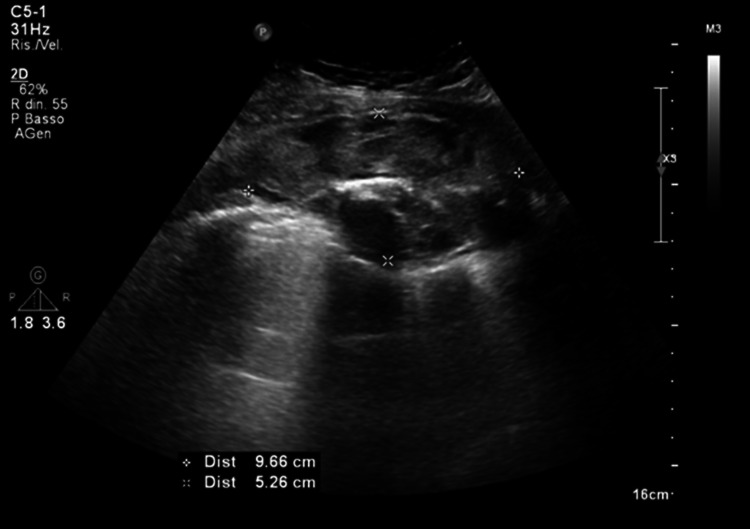
Image obtained by a convex probe on right hemitorax: there is an oval formation with an inhomogeneous echo pattern compatible with a hematoma, which occupies the plane of the intercostal muscles and exerts a mass effect on the underlying pleura

**Fig. 2:**
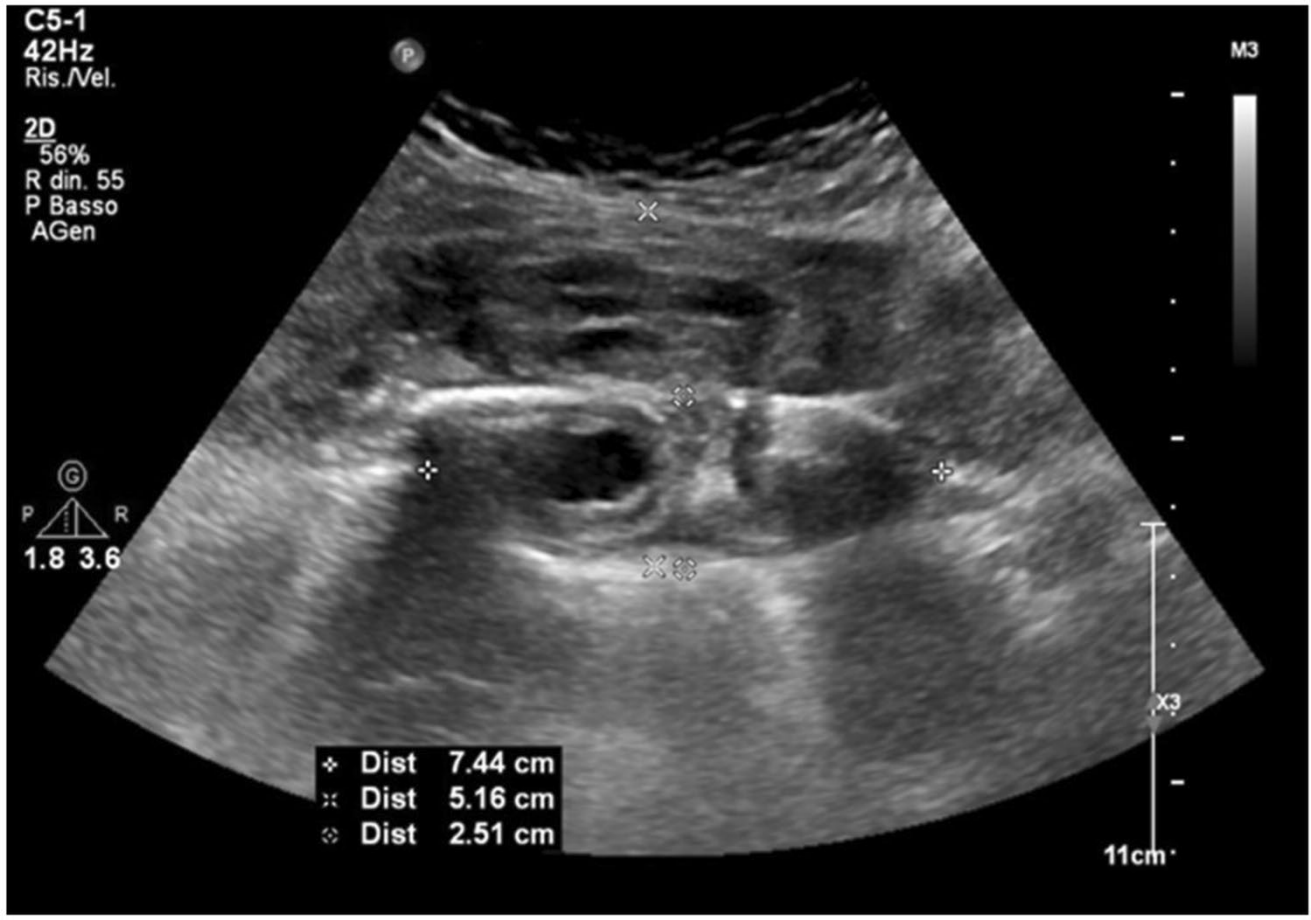
Image obtained by convex probe on right hemitorax: the hematoma exerts a mass effect on the pleura and has an anechoic oval formation in the lower part

Due to the appearance of swelling in the right hemithorax, persistence of pain and occurrence of fever, the patient performed a second access to the emergency room, where a chest computed tomography (CT) with contrast medium was performed thus showing a large hematoma of the pectoralis minor, some small air coefficients and possible lung contusion. On blood exams, C-reactive protein (CRP) and procalcitonin (PCT) were increased. Antibiotic therapy with piperacillina-tazobactam was prescribed. During hospitalization, clindamycin was added due to an increase of inflammation indices, and a surgical evaluation was request which did not place any operative indications. Blood microbiological culture resulted positive for methicillin-sensitive staphylococcus aureus. Therefore, the antibiotic therapy was modified by switching to oxacillin 3 g QID. The patient was transfused with two units of packed red blood cells due to progressive and persistent anemia (hemoglobin up to 7.6 g/dL). Thus, an ultrasound reevaluation was required (Figs. 1 and 2).

The ultrasound with color-doppler function and contrast enhanced ultrasound (CEUS) examination showed the appearance of active blood supply of the hematoma, and a small arterial bleeding of 6 mm was described (Videos 1 and 2).

This finding was suggestive for a pseudoaneurysm of the internal right mammary artery. Subsequently, the patient was evaluated by the interventional radiologist which indicated an attempt at selective embolization. Unfortunately, the embolization was unsuccessful due to the impossibility of selectively cannulating the affected branches. Ultrasound-guided injection of thrombin was carried out until complete interruption of the flow within the formation. At subsequent follow-up, no arterial or venous blush was highlighted. Due to the clinical stability the patient was discharged with imaging follow-up.

## Discussion

Mammary artery pseudoaneurysm is a rare complication of blunt chest trauma. Indeed, no more than 60 cases have been reported in the literature [10]. The main causes of traumatic IMA injuries are motorcycle and automobile accidents. Most frequently, these types of pseudoaneurysm are linked to iatrogenic complications from surgeries such as sternotomies, coronary artery bypass grafting, pacemaker placement, and central venous access [11, 12]. Diagnosis is often made with a contrast-enhanced chest CT scan, and later eventually confirmed with angiography.

In general, CT with contrast medium of the thorax is the reference exam in hemodynamically stable patients, which can detect active extravasation of contrast material.

Albeit digital subtraction angiography has long been the gold standard for the detection of active bleeding, CT accurately demonstrates the anatomic location of bleeding and indicates the vascular origin. Moreover, multidetector CT angiography provides a time efficient method for directing and planning therapy for patients with acute bleeding. CT is employed as a guide for angiographic or surgical intervention [13]. The additional information provided by multidetector CT angiography leads to faster selective catheterization of bleeding vessels, thus facilitating embolization.

The therapy is essentially based on different options such as endovascular treatment, surgery, percutaneous therapy and ultrasound-guided external compression therapy.

In our case, patient was treated by ultrasound-guided injection of thrombin. The success rates for embolization and surgically managed were 91.6% and 66.0%, respectively [14]. If a bleeding source is identified, super-selective catheterization followed by transcatheter micro-coil embolization is usually the most effective technique to control bleeding minimizing potential complications [15]].

## Conclusions

In trauma patients it is relevant to carry out continuous re-evaluations; ultrasound is an invaluable tool in performing a reevaluation. Ultrasound can be a useful tool for a specific and fine analysis of a given pathological finding such as hematoma. CEUS allows a detailed description of any active vascular supply in the context of hematoma.

### Supplementary Information

Below is the link to the electronic supplementary material.

Video 1. In the context of the hematoma, a small vessel is highlighted with a high-velocity turbulent color Doppler flow (aliasing) directed towards the anechoic space present in the lower part of the hematoma (MP4 1270 kb)

Video 2. The anechoic space in the lower part of the hematoma is in clear connection with the vessel previously indicated by the color Doppler, and it takes contrast medium in the arterial phase (15 seconds after the infusion); moreover, an arterial blood flow is clearly visible (MP4 7274 kb)

## References

[1] Boccatonda A, Grignaschi A, Lanotte AMG, Cocco G, Vidili G, Giostra F (2022). Role of lung ultrasound in the management of patients with suspected sars-cov-2 infection in the emergency department. J Clin Med.

[2] Boccatonda A, Cocco G, D’Ardes D, Delli Pizzi A, Vidili G, De Molo C (2023). Infectious pneumonia and lung ultrasound: a review. J Clin Med.

[3] Boccatonda A, Ianniello E, D’Ardes D, Cocco G, Giostra F, Borghi C (2020). Can lung ultrasound be used to screen for pulmonary embolism in patients with SARS-CoV-2 pneumonia?. Eur J Case Rep Intern Med.

[4] Montoya J, Stawicki SP, Evans DC, Bahner DP, Sparks S, Sharpe RP (2016). From FAST to E-FAST: an overview of the evolution of ultrasound-based traumatic injury assessment. Eur J Trauma Emerg Surg.

[5] Boccatonda A, Primomo G, Cocco G, D’Ardes D, Marinari S, Montanari M (2021). Not all abolished lung sliding are pneumothorax: the case of a particular lung atelectasis. J Ultrasound.

[6] Canelli R, Leo M, Mizelle J, Shrestha GS, Patel N, Ortega R (2022). Use of eFAST in patients with injury to the thorax or abdomen. N Engl J Med.

[7] Lichtenstein DA (2015). BLUE-protocol and FALLS-protocol: two applications of lung ultrasound in the critically ill. Chest.

[8] Wongwaisayawan S, Suwannanon R, Prachanukool T, Sricharoen P, Saksobhavivat N, Kaewlai R (2015). Trauma ultrasound. Ultrasound Med Biol.

[9] Cocco G, Ricci V, Villani M, Delli Pizzi A, Izzi J, Mastandrea M (2022). Ultrasound imaging of bone fractures. Insights Imaging.

[10] Chen JM, Lv J, Ma K, Yan J (2014). Assessment of internal mammary artery injury after blunt chest trauma: a literature review. J Zhejiang Univ Sci B.

[11] Toscano O, Bergonti M, Teruzzi G, Trabattoni D (2019). An unusual cause of suspected pulmonary hypertension: iatrogenic left internal mammary artery pseudoaneurysm. JACC Cardiovasc Interv.

[12] Falconieri F, Raevsky E, Davies S, Moat N (2015). Pseudoaneurysm of a branch of left internal mammary artery: a late and potentially fatal complication after redo-sternotomy. Interact Cardiovasc Thorac Surg.

[13] Shanmuganathan K, Mirvis SE, Sover ER (1993). Value of contrast-enhanced CT in detecting active hemorrhage in patients with blunt abdominal or pelvic trauma. AJR Am J Roentgenol.

[14] Whigham CJ, Fisher RG, Goodman CJ, Dodds CA, Trinh CC (2002). Traumatic injury of the internal mammary artery: embolization versus surgical and nonoperative management. Emerg Radiol.

[15] Walker PF, Daniel WT, Moss E, Thourani VH, Kilgo P, Liberman HA (2013). The accuracy of transit time flow measurement in predicting graft patency after coronary artery bypass grafting. Innovations (Phila).

